# Comparative Analysis of SiMoA and Ella Immunoassay Platforms for Measuring Serum Neurofilament Light Chain Levels in ATTRv With Polyneuropathy and Presymptomatic Carriers

**DOI:** 10.1111/ene.70215

**Published:** 2025-06-03

**Authors:** Guido Primiano, Marco Luigetti, Delia Righi, Angela Romano, Luca Leonardi, Valeria Guglielmino, Francesca Forcina, Marco Ceccanti, Maurizio Inghilleri, Fiore Manganelli, Stefano Tozza, Maria Ausilia Sciarrone, Francesca Vitali, Andrea Sabino, Carlo Manco, Angela Stufano, Maria Laura Stromillo, Nicola De Stefano, Paolo Calabresi, Domenico Plantone

**Affiliations:** ^1^ Dipartimento di Neuroscienze, Organi di Senso e Torace Fondazione Policlinico Universitario Agostino Gemelli IRCCS Rome Italy; ^2^ Dipartimento di Neuroscienze Università Cattolica del Sacro Cuore Rome Italy; ^3^ Department of Medicine, Surgery and Neuroscience University of Siena Siena Italy; ^4^ Neuromuscular and Rare Disease Centre, Neurology Unit Sant'Andrea Hospital Rome Italy; ^5^ Dipartimento di Neuroscienze, Salute Mentale e Organi di Senso (NESMOS) Sapienza Università di Roma Rome Italy; ^6^ Dipartimento di Neuroscienze Umane Sapienza Università di Roma Rome Italy; ^7^ IRCCS Neuromed Pozzilli Italy; ^8^ Department of Neuroscience, Reproductive and Odontostomatological Science University of Naples 'Federico II' Naples Italy; ^9^ Interdisciplinary Department of Medicine University of Bari Aldo Moro Bari Italy

**Keywords:** biomarker, ella, method comparison, neurofilament light chain, SiMoA

## Abstract

**Background:**

Neurofilament light chains (NfL) represent reliable serum biomarkers of neuroaxonal injury. Due to their low serum levels, precise detection methods are critical. This study aimed to scrutinize the comparability of two techniques: Single Molecule Array (SiMoA) and Ella automated immunoassay, analyzing serum NfL levels in ATTRv presymptomatic subjects and polyneuropathy patients.

**Methods:**

Blood samples were processed and analyzed using commercial Ella and SiMoA kits. Statistical analysis included the Wilcoxon signed‐rank test, Spearman correlation, Bland–Altman, and Passing‐Bablok regression. ANCOVA models were used to compare NfL levels between cohorts.

**Results:**

The study measured serum NfL levels in 55 symptomatic and 55 presymptomatic ATTRv subjects. Median NfL concentrations were significantly higher with Ella (median 27.5 pg/mL) than SiMoA (median 15.9 pg/mL). Both methods showed a strong positive correlation (*r* = 0.8, *p* < 0.001), but Ella overestimated NfL by 42%. Bland–Altman analysis revealed a mean bias of 15.4 pg/mL, with limits of agreement between −41.1 and 72.0 pg/mL. The slope of the Passing–Bablok regression line was 0.58, and the intercept was 3.48 pg/mL, suggesting that the Ella platform tends to overestimate NfL concentrations compared to the SiMoA platform, especially at higher concentrations. Both methods effectively distinguished presymptomatic from symptomatic patients (*p* < 0.001 for both).

**Conclusions:**

Our findings underscore that both platforms are effective in measuring serum NfL, with Ella consistently overestimating, especially at higher concentrations. The difference between the two platforms must be taken into account when deeming the concentrations as pathological or normal, as well as when conducting longitudinal studies.

## 1 Introduction

Neurofilament light chain (NfL) is a neuron‐specific cytoskeletal protein present in the cell bodies and axons of neurons, playing a crucial role in structural support and axonal polarization [1, 2]. Over the past few years, NfL has been extensively studied for its significance as a non‐specific marker of axonal damage. With the development of highly sensitive methods, it has become possible to measure its concentrations in biological fluids, particularly cerebrospinal fluid (CSF) and peripheral blood, with good precision and reproducibility [3, 4]. Currently, the measurement of NfL has assumed important diagnostic and monitoring value for patients affected by various neurodegenerative conditions [5]. Recently, NfL has been included as a biomarker of neurodegeneration in the diagnostic criteria for Alzheimer's disease [6], while in multiple sclerosis, regular monitoring of serum NfL levels has been proposed for its high prognostic value for subclinical disease activity, relapse risk, and the development of gadolinium‐enhancing lesions [7]. About peripheral nervous system pathologies, NfL levels are also used as biomarkers of neuroaxonal damage. NfL has been demonstrated to increase in patients with acute [8] and chronic [9] demyelinating polyradiculoneuropathy, diabetic polyneuropathy [10], and with neurogenetic conditions such as hereditary amyloidogenic transthyretin (ATTRv) amyloidosis with polyneuropathy [11, 12, 13, 14]. ATTRv is a rare, progressive, multisystem disorder of adult onset that is ultimately fatal. It is caused by specific pathogenic variants in the transthyretin (*TTR*) gene. The disease demonstrates variable penetrance and phenotypic heterogeneity, even among individuals harboring the same genetic variant [15].

However, the existing literature on this topic reveals a heterogeneous landscape, particularly in relation to the diverse ultrasensitive techniques employed by different research centers for the detection of NfL. For certain central nervous system disorders, comparative analyses of multiple diagnostic or assessment methodologies have already been undertaken [16, 17, 18, 19].

Currently, the two most widely used commercial platforms in clinical research for measuring NfL in blood are the Single Molecule Array (SiMoA, Quanterix Inc., Lexington, MA, USA) and Ella (ProteinSimple, Bio‐techne, Minneapolis, MN, USA) platforms. SiMoA is a digital immunoassay technology that uses non‐competing monoclonal antibodies and arrays of microwells to detect single molecules, offering extremely high sensitivity. Ella, on the other hand, is an immunoassay platform based on microfluidic cartridges that allows the automated and high‐throughput quantification of soluble biomarkers.

Despite the growing importance of NfL as biomarkers, there are few direct comparisons between the two platforms, especially in specific clinical contexts such as peripheral neuropathy. To the best of our knowledge, no studies are comparing the two methods in patients with peripheral nervous system diseases. Therefore, this study aims to compare the performance of SiMoA and Ella in measuring serum NfL levels in symptomatic ATTRv subjects and presymptomatic ATTRv subjects, where NfL has been demonstrated to represent a reliable biomarker of nerve damage and polyneuropathy [20], to evaluate the correlation between the two methods and their reliability in detecting NfL levels in peripheral neuropathy.

## 2 Method

### 2.1 Study Subject

This multicentre, cross‐sectional cohort study included subjects with confirmed pathogenic *TTR* variants, including both symptomatic ATTRv subjects and presymptomatic ATTRv subjects. The enrolled subjects are routinely followed at the renowned Italian reference centers for ATTRv, with long experience in the clinical and genetic diagnosis of this genetic disorder. All enrolled subjects underwent an extensive medical evaluation, as previously described [14]. Presymptomatic subjects were identified as individuals carrying the genetic variants associated with ATTRv but exhibiting no clinical symptoms or instrumental signs of polyneuropathy. Clinical evaluations were conducted by a neurologist experienced in peripheral nervous system diseases. Symptomatic ATTRv subjects with polyneuropathy were defined based on the presence of clinical symptoms of peripheral neuropathy, with the diagnosis supported by abnormal findings in electrophysiological studies, including traditional nerve conduction studies and Sudoscan. All symptomatic ATTRv subjects with polyneuropathy had genetic confirmation of the *TTR* variant and a clinical history consistent with disease progression. Confirmation of amyloid deposits was not required in our population of late‐onset patients, as in our centers, to establish disease onset [21].

### 2.2 Ethics Statement

The study adhered to the principles outlined in the 1964 Declaration of Helsinki and its subsequent revisions. The study was approved by the Comitato Etico Territoriale Lazio Area 3 at the Fondazione Policlinico Agostino Gemelli IRCCS (**protocol ID 5470**).

### 2.3 Serum Collection

Peripheral blood was collected in additive‐free vacutainers containing separating gel. It was then.

centrifuged at 3000 rpm for 10 min at room temperature to separate the serum, which was.

subsequently collected into sterile polypropylene tubes and stored at −80° Celsius.

### 2.4 NfL Assay (SiMoA)

Serum NfL concentrations were assessed in each of 55 ATTRv symptomatic subjects and 55 pre‐symptomatic subjects using the commercially available immunoassay kits for NfL (Quanterix, Billerica, MA, USA). The assay was run on the semi‐automated ultrasensitive SR‐X Biomarker Detection System (Quanterix). Samples were diluted 1:4 and randomly distributed on 96‐well plates. Quality control (QC) samples, provided with the kit, exhibit concentrations in the predefined range, and the coefficient of variance between plates was maintained below 10%. All samples were analyzed in a blinded manner using alphanumeric codes. Diagnostic codes were revealed only after QC‐verified NfL concentrations were reported to the database manager. Concentrations were measured in pg/ml and documented in the database. Regarding the detection range for serum NfL concentration values, the SiMoA platform allows a lower limit of detection of 0.071 pg/mL and an upper limit of detection of 2000 pg/mL. The analyses were conducted at the Centre for Precision Medicine and Translation laboratory of the University of Siena, Italy.

### 2.5 NfL Assay (Ella)

Serum NfL concentration was measured in each of 55 symptomatic ATTRv subjects and 55 presymptomatic subjects using the Simple Plex cartridge‐based assay on the Ella platform (ProteinSimple, San Jose, CA, USA). Ella was calibrated using the in‐cartridge factory standard curve. All samples were measured on the same day, with a 1:2 dilution factor, according to the manufacturer's instructions. Regarding the detection range for serum NfL concentration values, the Ella platform allows a lower limit of detection of 1.09 pg/mL and an upper limit of detection of 10,290 pg/mL. The analyses were conducted at the Fondazione Policlinico Agostino Gemelli IRCCS, Rome, Italy.

### 2.6 Statistical Analysis

Descriptive analyses of demographics were performed by calculating median and 25th –75th percentiles.

NfL concentrations obtained by Ella and SiMoA methods were compared using the Wilcoxon signed‐rank test. The Spearman correlation coefficient was calculated to examine the association between serum NfL concentrations from SiMoA and Ella within the same sample series, with a 95% confidence interval reported.

The Bland–Altman method [22] was used to evaluate the mean difference and the 95% limits of agreement between NfL concentrations measured using the SiMoA and Ella methods. Subsequently, the Bland–Altman analysis was repeated on NfL concentrations obtained with both methods, grouping the data based on a cut‐off applied to NfL concentrations measured with the Ella method. The cut‐off was defined as the mean of the medians of the concentrations from both methods. The overestimation/underestimation in percentage was calculated for each assessment as [(mean bias/NfL concentrations mean of the two methods) × 100]. Finally, the regression relationship was evaluated using Passing–Bablok [23]. The Passing–Bablok regression is a non‐parametric method used to compare two measurement methods (in our case, the SiMoA and Ella platforms). It is particularly useful when the data do not meet the normality requirements. In Passing–Bablok regression, two parameters are considered: the slope of the regression line, which serves as an index of the relationship between the variations in the two measurement methods (the further the slope deviates from 1, the more it indicates that one method tends to produce proportionally higher or lower values than the other), and the intercept (the further the intercept is from 0, the more it indicates that one method tends to produce consistently higher or lower values than the other at lower concentrations).

The non‐normal distribution of the NfL measurements obtained from the SiMoA and Ella platforms was evaluated using the Shapiro–Wilk test, and logarithmic transformations (Log10) were applied when the normality assumption was violated, following published studies [24] when comparing concentrations between presymptomatic and symptomatic ATTRv subjects. Differences in serum NfL levels between groups were evaluated using analysis of covariance (ANCOVA) models, adjusting for age.

Statistical significance was defined as a *p*‐value of less than 0.05. Statistical analyses were conducted using Jamovi Software (The Jamovi Project, 2021), and MedCalc Software (MedCalc Software, Ostend, Belgium).

## 3 Result

**FIGURE 1:**
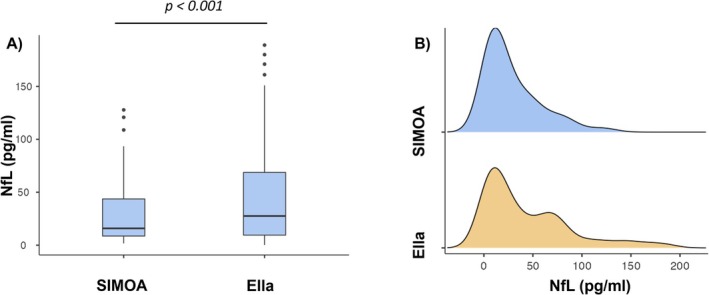
Boxplots and histograms with density curves. (A) Box plots express the first (Q1) and third (Q3) quartiles by the upper and lower horizontal lines in a rectangular box, in which there is a horizontal line showing the median. The whiskers extend upwards and downwards to the highest or lowest observation within the upper (Q3 + 1.5 × IQR) and lower (Q1–1.5 × IQR) limits. *p* values indicate statistical significance between the different groups. (B) histograms with density curves showing the comparison of NfL concentrations distribution measured by SiMoA and ELLA methods.

Serum NfL concentrations were measured in 55 symptomatic and 55 presymptomatic subjects using SiMoA and Ella. The demographics of the whole recruited cohort and of the ATTRv presymptomatic subjects and symptomatic subjects are reported in Table 1, including *TTR* variants of the ATTRv cohort. In the whole ATTRv cohort, the median serum NfL concentration was 15.9 pg/mL (25th–75th percentile 8.62–43.7) with SiMoA and 27.5 pg/mL (25th–75th percentile 9.46–68.7) with Ella, with significantly higher concentrations measured by Ella (W = 1024.0; *p* < 0.001) than by SiMoA (Figure 1).

**TABLE 1:** Descriptive characteristics of patients' demographic data and *TTR* variants.

	Whole cohort	Pre‐symptomatics subjects	Symptomatics subjects
Sex (Female/tot)	41/110	28/55	12/55
Age (median, 25th Percentile – 75th percentile)	64 (47–72)	47 (42,5–58)	71 (66,5–75)
Mutation (number, mutation type, number of females)		30 V30M, 16F 14 F64L, 6F 3 I64L, 3F 3 V122I, 0F 3 E89Q, 2F 1 A109S, 1 F 1 E62K, 0F	27 V30M, 3F 16 F64L, 6F 4 I64L, 0F 3 V122I, 0F 2 E89Q, 2F 1 A109S, 0 F 1 A120S, 1F 1 V32R, 0F

Moreover, SiMoA and Ella serum NfL concentrations showed a strong positive correlation (*r* = 0.8, *p* < 0.001) in the whole ATTRv cohort.

**FIGURE 2:**
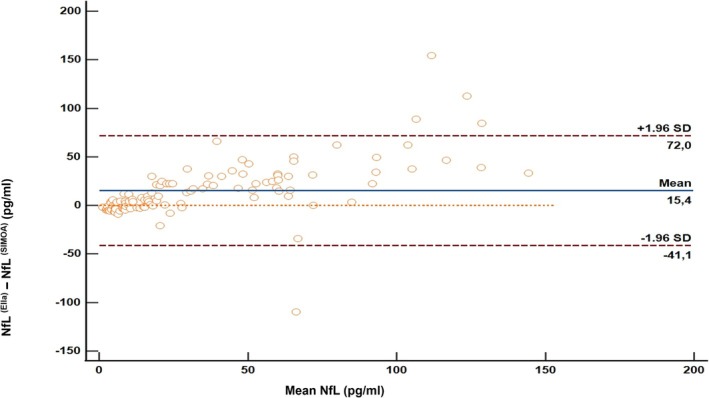
Bland–Altman plots illustrating the agreement between the two measurement methods. The X‐axis represents the mean values of the two methods, while the Y‐axis represents their differences. The blue central line indicates the mean bias, the upper and lower dashed lines correspond to the limits of agreement (±1.96 SD).

The Bland–Altman analysis revealed a mean bias of 15.4 pg/mL (*p* < 0.001), with Ella showing a 42% overestimation compared to SiMoA. The limits of agreement (LOA) were as follows: lower limit −41.1 pg/mL (95% CI [−50.4, −31.7]) and upper limit 72.0 pg/mL (95% CI [62.6, 81.3]), with 95% of observations falling within these limits (Figure 2).

**FIGURE 3:**
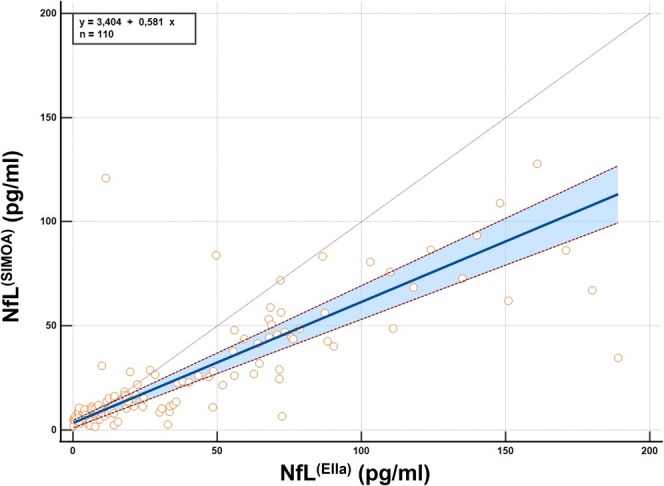
Passing Bablock regression plot comparing NfL measurements between Ella and SiMoA methods. The x‐axis represents NfL concentrations measured by ELLA (pg/ml), and the y‐axis represents NfL concentrations measured by SiMoA (pg/ml). The blue line indicates the linear regression fit, with the shaded area representing the confidence interval. The equation of the regression line is y = 3.4 + 0.58x, and the sample size is *n* = 110.

The regression line equation is (y = 3.404 + 0.581x), where (x) represents the NfL concentrations measured with the Ella platform and (y) represents those measured with the SiMoA platform. The intercept of 3.404 pg/mL indicates the value on the y‐axis (SiMoA) when the x‐axis (Ella) is zero. This suggests that, even when the Ella platform measures a concentration of zero, the SiMoA platform would still report a concentration of 3.404 pg/mL. This positive intercept implies a systematic bias where the SiMoA platform tends to report higher values compared to the Ella platform at low concentrations. Additionally, the slope of 0.581 indicates that for every unit increase in the concentrations measured with Ella, the concentrations measured with SiMoA increase by 0.581 units. This slope value less than 1 suggests that the Ella platform tends to overestimate NfL concentrations compared to the SiMoA platform, especially at higher concentrations. The 95% CI of intercept and slope values differ from zero and one, respectively, indicating a method agreement. Moreover, the linearity test demonstrated no significant deviation from linearity between the two datasets (*p* = 0.44), suitable for concluding on method agreement (Figure 3) [16, 17, 18, 19, 20, 22, 23, 24, 25].

**FIGURE 4:**
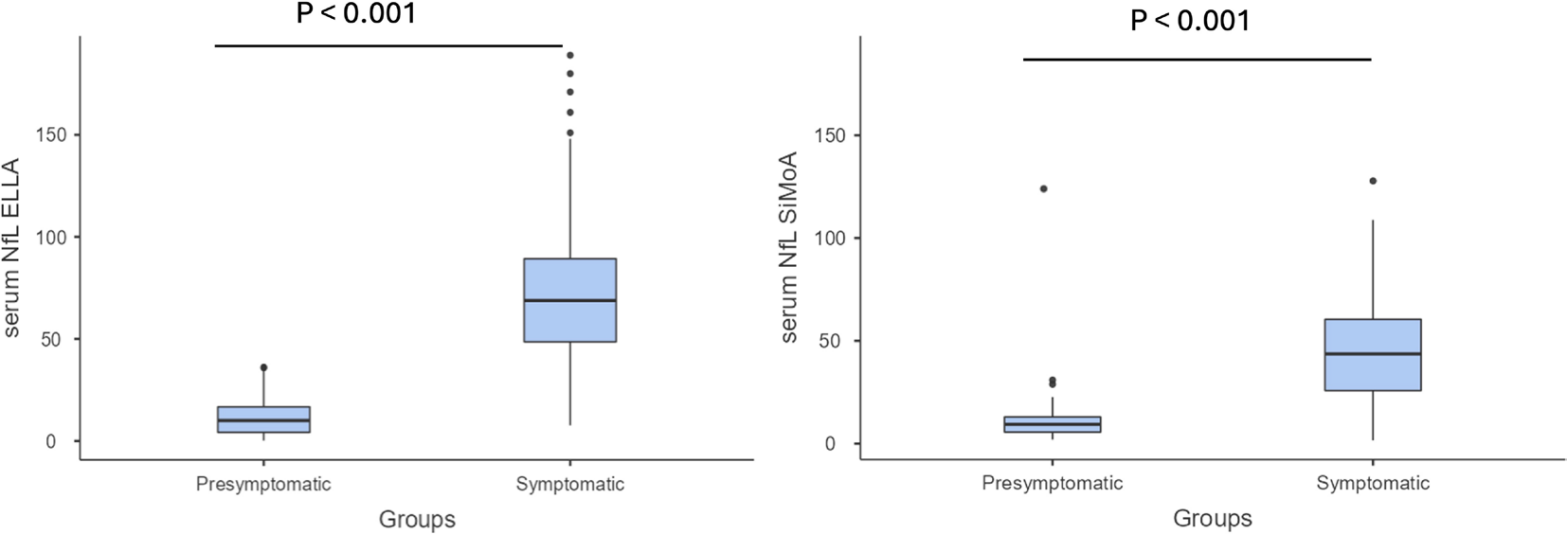
Comparison of serum NfL levels between presymptomatic subjects and symptomatic ATTRv patients using both Ella and SiMoA.

Serum NfL concentrations were significantly higher in symptomatic ATTRv patients compared to presymptomatic subjects (Figure 4) using both Ella (*p* < 0.001 median log10 serum NfL in presymptomatic subjects 0.99, 25th–75th percentile 0.62–1.22; median log10 serum NfL in symptomatic ATTRv patients 1.84, 25th–75th percentile 1.69–1.95) and SiMoA (*p* < 0.001 median log10 serum NfL in presymptomatic subjects 0.97, 25th–75th percentile 0.74–1.11; median log10 serum NfL in symptomatic ATTRv patients 1.64, 25th–75th percentile 1.41–1.78).

## 4 Discussion

The findings of this study highlight the comparative performance of two widely used immunoassay platforms, SiMoA and Ella, in measuring serum NfL levels in symptomatic ATTRv subjects with polyneuropathy and presymptomatic subjects harboring *TTR* variants. The importance of measuring serum NfL concentrations has been widely demonstrated in numerous conditions affecting the peripheral nervous system, including ATTRv polyneuropathy, where such measurements have been shown to distinguish symptomatic patients from presymptomatic subjects [14] and to be useful for monitoring the clinical progression of the disease [26].

Our study shows that both methodologies are valid in detecting NfL serum concentrations, with a strong positive correlation (*r* = 0.8, *p* < 0.001) between them, confirming their reliability. Remarkably, the slope of the Passing–Bablok regression line was 0.58, confirming previous studies that have conducted this comparison in central nervous system diseases. However, the linearity between the two methods appears to diminish at higher NfL concentrations. The observed non‐linearity at higher concentrations may explain the higher increment factor observed with the Ella platform in our study compared to prior analogous investigations [16, 17, 18, 19]. Indeed, our study demonstrated a more pronounced elevation in NfL concentrations measured using the Ella platform compared to SiMoA, with values approximately 40% higher. This difference exceeds the 15%–25% increase reported in previous studies that assessed the same comparison across various biofluids, including cerebrospinal fluid (CSF), serum, and plasma [16, 17, 18, 19]. The amplified discrepancy observed in the present study may be attributed to the distinct characteristics of our patient cohort, which included a higher proportion of individuals exhibiting elevated levels of neuroaxonal damage relative to cohorts examined in prior studies. Previous investigations have predominantly focused on patients with multiple sclerosis [16, 17, 19], and to a lesser extent on individuals with dementia and healthy controls [18]. In contrast, the present study is the first to conduct these analyses exclusively in patients exhibiting neuroaxonal damage resulting from peripheral nerve injury.

Another aspect to discuss is the intercept of 3.404 pg/mL, which suggests a systematic bias with an overestimation of the SiMoA platform compared to the Ella platform at low concentrations. This bias may be attributed to the higher sensitivity of SiMoA at lower concentrations, a distinction further supported by the differing lower limits of detection between the two platforms.

As expected, both methodologies exhibited comparable performance in differentiating presymptomatic ATTRv subjects from symptomatic individuals (*p* < 0.001 for both). Therefore, although both methodologies are valid, their key distinction resides in the lower limit of detection, quantified at approximately 2.7 pg/mL for Ella and 0.1–0.2 pg/mL for SiMoA [27, 28] and the use of different calibrators. Ella employs naturally derived bovine NfL, whereas SiMoA uses recombinant human NfL [29]. This difference in calibration standards could account for the systematic bias, as the antibodies used in the assays may have varying affinities for the different NfL proteins, leading to differential detection sensitivities [16]. Moreover, it is not possible to exclude the potential impact of different dilution factors (1:2 for Ella and 1:4 for SiMoA) on the variability of the concentrations obtained with the two platforms. However, for routine clinical purposes, it should be acknowledged that this difference between the two platforms has a limited impact, in that both Ella and SiMoA can easily differentiate patients with and without neuroaxonal damage.

The identification of absolute cut‐off values for sNfL concentrations in our study is not feasible, as normal values for sNfL concentrations vary significantly with age, making it challenging to establish a single cut‐off applicable to all patients. Moreover, the development of normative data and specific cut‐off values extends beyond the scope of this work, which primarily focuses on comparing the Simoa and Ella platforms for measuring NfL levels. For the Simoa method, reference cut‐off values and normative data are already available elsewhere (e.g., https://shiny.dkfbasel.ch/baselnflreference/?code=a3pex2vx) [30].

Our study has some limitations. Although the measured values in our study did not reach the upper detection limits of the two methods, the inclusion of patients with a peripheral nervous system pathology, characterized by increased NfL concentrations in its symptomatic phase, should still provide a representative range of concentrations for this population. However, additional studies could provide a more comprehensive comparison of the two platforms in other neurological disorders typically associated with elevated serum NfL concentrations, including amyotrophic lateral sclerosis and specific types of dementia, and provide a better generalizability of the results. Second, this study is cross‐sectional and does not include longitudinal data, which could provide important insights into how and over what time periods serum NfL concentrations vary. Future longitudinal studies will be necessary to understand how the two platforms perform in monitoring NfL levels during disease progression or in response to treatments. In addition, our study did not evaluate the spike recovery rates and matrix effects for either the SiMoA or Ella platforms. These factors may have the potential to impact the accuracy of NfL measurements, as variations in recovery and matrix interference might affect the results. Future studies should consider evaluating these parameters to enable a more thorough assessment of the performance and reliability of these assays in complex biological samples. A final limitation of our study pertains to the fact that measurements were performed by different operators in different laboratories. This approach was necessitated by logistical constraints and the high cost of the devices used.

Our study demonstrates that both Ella and SiMoA represent reliable methodologies for the serum assay of NfL in patients with peripheral polyneuropathy, although they are not interchangeable. However, the methodology employed must always be considered when interpreting the obtained concentrations, as the same concentration may be classified as either normal or pathological depending on the method utilized. The methodology must also be considered in longitudinal studies, as the use of different assay methods may lead to artificial increases or decreases in serum NfL concentrations that do not reflect actual changes in neuroaxonal damage.

## 5 Conclusion

In conclusion, while both SiMoA and Ella are effective for measuring serum NfL, the consistent overestimation observed with Ella, particularly at higher concentrations, underscores the importance of considering the method used when interpreting the results. Future research should prioritize the development of standardized conversion factors to address these discrepancies, thereby ensuring more accurate and comparable NfL measurements across various clinical settings and patient populations. Additionally, further studies are warranted to explore the potential impact of these differences on the diagnostic and prognostic utility of NfL as a biomarker in various neurological and peripheral nerve disorders.

## Author Contributions

All the authors provided critical feedback and approved the final manuscript. Conceptualization: D.P., M.L., G.P., A.R., L.L.; Writing and Original Draft: D.R., C.M., A.R.; Writing and Review and Editing: D.P., G.P., M.L., V.G., F.F., M.C., M.I., F.M., S.T., M.A.S., F.V., A.Sa., A.St.; Supervision: D.P., N.D.S., P.C.

## Conflicts of Interest

The authors declare no conflicts of interest.

## Data Availability

The data that support the findings of this study are available from the corresponding author upon reasonable request.
